# 

**Published:** 1916-04

**Authors:** 


### EDITORIAL DEPARTMENT

MRS. F. E. DANIEL, Publisher and Managing Editor.

### To Subscribers.

For your convenience and in order that your files may not be broken, your subscription is on a continuous basis, and you are responsible for it until you notify us to discontinue. A notice to the postmaster is not sufficient; that may never reach us.

Another thing: When you received your subscription bill recently did you attend to it at once, or did you put it away in your desk and promptly forget it? It was only a small amount and you are going to pay it. Why not attend to it at once? If you will reflect just a moment on the time, labor and expense of sending out several thousand bills, you will not wait for a second or third bill to be sent you.

Don't you feel like giving your very best thought and care to the patient who pays his bills promptly and cheerfully? Same here; the editor is human.

### Deaths.

Dr. James H. Stiver of Denison died at the home of his daughter March 11th.

Dr. S. H. Dickerson, a prominent physician of Chandler, was almost instantly killed on February 28th when his car turned completely over with him, pinning him under. His skull was crushed. He only lived ten minutes after the accident.

A two-year fight to delay death that discoveries of value to medicine might be made, was lost in the death of Dr. William C.

Bouton of Waukegon, Ill., on March 1st. The doctor was afflicted with an incurable form of paralysis, and hoped to live long enough to discover in his disease something of value to the world.

Dr. J. E. Brown of McGregor died at his home on March 12th after a lingering illness of many months.

Dr. Aaron Suhler of Waco died March 1st.

Dr. Alfred Patten of Mineola, one of Wood county's oldest and wealthiest citizens, died March 7th after an illness of two months.

Dr. U. M. Gilder of Gatesville, who lived alone, was found dead at his home March 6th.

Dr. Charles S. Powell of Benton, Ariz., died in El Paso March 18th, where he had been taken for treatment.

Dr. Holmsley of Highland, Erath county, died recently.

Dr. John W. Davis of Itasca dropped dead at his home on March 13th.

Dr. Wm. L. Rodman, president of the American Medical Association and professor of surgery at Medico-Chirurgical College, Philadelphia, died March 8th.

Dr. Granderson R. Sorrels, a retired physician, died March 21st at the home of his son in Dallas.

The body of Dr. Carlos Husk, who died of typhus at Laredo, recently was taken to El Paso for burial.

Dr. A. L. C. Hindsman died at his home in Fort Worth on March 19th.

Dr. Alex Irvin of Wallis, Texas, died at his home March 24th. He served five years in the Confederate army, and had been a practicing physician since.

